# Comparative Analysis of the Status and Influencing Factors of Immunization Among Children Between Registered and Floating Population

**DOI:** 10.3389/fpubh.2022.872342

**Published:** 2022-06-09

**Authors:** Yan Xiong, Yaqing Xue, Guojin Jiao, Jun Xie, Jingmin Cheng

**Affiliations:** ^1^School of Public Health, Shanxi Medical University, Taiyuan, China; ^2^School of Management, Shanxi Medical University, Taiyuan, China; ^3^School of Public Health, Southern Medical University, Guangzhou, China; ^4^School of Health Management, Southern Medical University, Guangzhou, China; ^5^School of Basic Medicine, Shanxi Medical University, Taiyuan, China

**Keywords:** child health, vaccination, basic public health services, registered population, floating population, comparative analysis

## Abstract

**Background:**

A vaccine is an effective tool to reduce the gap between the rich and the poor and improve health equity, through which a number of serious childhood diseases can be successfully prevented or eradicated. This study is aimed to compare the current situation of vaccination and related factors among children in China's registered residents and floating population, to provide a reference for achieving the 100% vaccination rate in China.

**Methods:**

The data used for this study are from the 2017 National Migrants Dynamic Monitoring Special Survey data. A self-designed questionnaire was used to collect information, such as socio-demographics, vaccination status of children, and so on, on the registered population and floating population. Descriptive statistics and a chi-square independence test were used to describe the information and to compare the vaccination status of children under different sociodemographic characteristics. Binary logistic regression was employed to analyze influencing factors associated with vaccination of children.

**Results:**

The findings showed that 94.39% of children in registered residence were completely vaccinated, which was significantly higher than that of the floating children (91.68%, *p* < 0.001). The region, parents' education level, and marital status were found to be significant risk factors for complete vaccination of children regardless of the registered or floating population. In addition, ethnicity and length of time to the nearest medical institution were unique risk factors for complete vaccination of children in registered residence. And, health record was an independent influencing factor for vaccination of children of floating population.

**Conclusion:**

Compared with registered population, floating population was at a disadvantage in using basic public health services, especially in children's vaccination. To achieve 100% vaccination for children, particular interventions should be taken for different populations.

## Introduction

Population mobility is a special and important social phenomenon in the process of China's economic and social modernization. Since the reform and opening up, with the transformation of the economic system and the rapid development of urbanization, a large number of surplus laborers in rural areas have flowed into cities to work (1). In recent years, family migration has become a new trend in the migration process in China. That is, the migration trend has changed from “temporary residence” and “migrant alone” to “long-term residence” and “migration with core family members” (2). This means that floating children are increasingly becoming an important part of the floating population. However, long-term mobility exposes floating children to greater health risks (3). The vulnerability of the floating population is obvious in terms of livelihood insecurity, negligence, and alienation in the new sociocultural environment (4, 5). Studies have shown that the floating population is the high-risk susceptible population to infectious diseases, occupational diseases, chronic diseases, and psychological problems (6, 7). Therefore, how to solve the health inequality caused by population mobility are the problems and challenges faced by the current system promoters.

The health level in childhood not only affects a person's health status throughout the life but also relates to the education, employment, and income in adulthood. Considering the dual characteristics of mobility and children, the health status of floating children deserves more attention.

Immunization has shown to be one of the most cost-effective health interventions worldwide, through which a number of serious childhood diseases can be successfully prevented or eradicated (4). The implementation of immunization programs varies greatly in different countries and regions (8); however, the vaccination situation of floating children in some countries is relatively similar, i.e., the vaccination rate of floating children is generally low. Scholars have conducted empirical research studies on the related factors of children's vaccination and have drawn some conclusions. Studies have found that in addition to immigration or non-immigrant status factors, personal factors, such as parental education, occupation, knowledge, attitude, awareness of autonomous immunity, and family income, were significantly correlated with children's vaccination (9–12). In addition, social integration was found to be positively associated with floating children's vaccination status (13). Meanwhile, accessibility of vaccination services, vaccine supply, and health service policies also affected vaccination information. Are there any differences in the influence of these factors on the vaccination of registered residence children and floating children? At present, the research in this field is still relatively limited.

In addition, as an important part of basic public health services, health education is of great significance to maintain health and reduce the risk of poverty (14). Previous studies have found that there is a current situation of “three low and one high” in the immunization of floating children in China, i.e., low immunization rate, low card building rate, low awareness of parents, and high incidence rate of infectious diseases (15). It can be seen that parents' health education is closely related to children's immunization. Based on the above, the main aim of this study are as follows: first, to compare the vaccination situation of the children in the registered residence population and the floating population and analyze the current situation of the parents of the children who are vaccinated completely. Secondly, the difference in the factors affecting the vaccination of the registered residence children and the floating children is compared, focusing on the factors of parents' health education, to provide a scientific basis for promoting the health equity and improving the children's health.

## Methods

### Study Design and Participants

This study used data from the China Migrants Dynamic Survey (CMDS) in 2017, which was provided by the Migrant Population Service Center. CMDS is an annual national sample survey of the internal migrants organized by the National Health Commission (NHC), which aims to understand the changing landscape of internal migration, the utilization of public health services, and the management of family planning services (16). The survey is conducted in 32 provincial units, which cover all 31 provinces and the Xinjiang Production and Construction Corps (XPCCs) of China. In order to understand the epidemic status of key diseases, in addition to the original survey, 8 cities were selected for a special survey in 2017. This study is based on this survey. Sampling sites included Qingdao, Suzhou, Guangzhou, Zhengzhou, Changsha, Jiulongpo District, Urumqi, and Xishuangbanna. From the perspective of location, Qingdao, Suzhou, and Guangzhou are located in the east, which is more economically developed; Zhengzhou and Changsha belong to the central region; Jiulongpo District, Xishuangbanna, and Urumqi belong to the western region. The data were standardized to adjust for bias caused by differences between regions.

The participants were selected by using a stratified multi-stage sampling method with a probability-proportional-to-size (PPS) approach. First, 31 provinces (autonomous regions and municipalities) and XPCCs were taken as the first-level sample units, eight representative provinces were selected. Second, one city in each province was selected, as follows: Qingdao, Suzhou, Guangzhou, Zhengzhou, Changsha, Jiulongpo District, Urumqi, and Xishuangbanna. Then, in each selected city according to the administrative division, township (town, street) attributes were sorted, as the third layer. Next, selected townships (towns and streets) by the PPS method. In the selected township (town, street), the village (neighborhood) committee was selected by the same method. All eligible subjects in the selected village (neighborhood) committees were invited to participate in the study. In each village or neighborhood, floating populations' households were selected by simple random sampling according to a random number table. The floating population that lived in the destination for more than 1 month, aged 15 and over, and were not registered in the district (county or city) were included in the study. Similarly, the registered families were selected according to the same sampling method as the floating populations' households. The registered population aged 15 and above at each sampling point was included in the study. A household needs to investigate only one mobile population or registered residence population. Finally, a total of 13,998 floating population and 14,000 registered residence population were surveyed. Information collected included participants' basic information, family members, health and public services, social integration, and epidemic influencing factors of key diseases, etc.

### Dependent Variable

According to the research needs of this study, the dependent variable was children's vaccination. This variable was measured by the following question: “Has your child been vaccinated on time since birth?.” Possible answers were as follows: yes, no, and not applicable. We excluded all “not applicable” responses, resulting in a total of 12,199 participants included.

### Independent Variables

#### Socio-Demographics

Socio-demographic characteristics included the following: region, gender, age, ethnicity, education level, marital status, and chronic disease. The region was classified into the eastern region, central region, and western region. Education level was coded into four categories, namely, primary school or below, junior high school, senior high school, university or college, and above. Chronic diseases were measured through the question, “Do you suffer from chronic diseases diagnosed by doctors, such as hypertension or diabetes?.” The possible answer was “yes” or “no.”

#### Health Education

The health education was reflected by the question: “Have you received the following health education in your local community in the past year?.” The response options were “yes” and “no.” The types of health education mainly consisted of “occupational disease prevention and control,” “STD and AIDS prevention and control,” “reproductive health and contraception,” “tuberculosis prevention and control,” “tobacco control,” “chronic disease prevention and control,” “maternal and child healthcare,” “healthy birth and childbearing,” “self-help education in public emergencies,” and “mental health” education. Respondents should answer the question according to their utilization of health education. In this study, the respondents who have received any one of the above health educations are regarded as having received health education. In view of the delay of the floating population receiving the health education services in the inflow area, the floating population that has lived in the destination areas for <6 months will be excluded.

In addition, length of time to the nearest medical institution, health records, and cognition of basic public health services were included in this study. The length of time to medical institutions is mainly to evaluate the accessibility of individual medical services. The establishment of health records is also one of the contents of basic public health services.

### Statistical Analysis

Data were processed and analyzed using STATA version 14.0. Descriptive statistics and a chi-square independence test were used to describe the information and compare the vaccination status of children under different sociodemographic characteristics. To further examine potential factors associated with risk or protection for children vaccination, a binary logistic regression analysis was used, and odds ratios (ORs) and 95% CIs were calculated. All tests were 2-tailed, and statistical significance was set at the 5% level.

## Results

### Sample Characteristics

The characteristics of the participants are present in Table 1. Of the 12,199 participants, 49.82% (6,078) participants were registered population, 50.18% (6,121) participants were floating population. Among the registered population, 51.60% participants were men, and the mean age was 33.74 years (SD, 7.66). The majority of participants (87.46%) were of Han nationality. In total, 52.58% of the individuals had received an education of junior college or above and 95.97% were married. For the floating population, 50.60% were men, the mean age was 34.33 years (SD, 8.29), 88.83% were of Han nationality. Compared with the registered population, the education level of the floating population is relatively low, and only 19.02% had a college degree or above. Chi-square testing showed that there were significant differences in registered and floating populations in the regional classification, age, ethnicity, education level, marital status, length of time to the nearest medical institution, health records, chronic disease, and cognition of basic public health service (*p* < 0.05).

**Table 1:** Characteristics of participants (*N* = 12,199).

**Variables**	**Registered population**	**Floating population**	***P***
	***n*%**	***n*%**	
**Region**			<0.001
Eastern region	2,755 (45.33)	2,761 (45.11)	
Central region	1,690 (27.80)	1,358 (22.19)	
Western region	1,633 (26.87)	2,002 (32.70)	
**Gender**			0.269
Male	3,136 (51.60)	3,097 (50.60)	
Female	2,942 (48.40)	3,024 (49.40)	
**Age (years)**			0.010
≤ 30	2,484 (40.87)	2,361 (38.57)	
>30	3,594 (59.13)	3,760 (61.43)	
**Ethnicity**			0.020
Han ethnic	5,316 (87.46)	5,437 (88.83)	
Minorities	762 (12.54)	684 (11.17)	
**Education level**			<0.001
Primary school or below	226 (3.72)	747 (12.20)	
Junior high school	1,063 (17.49)	2,585 (42.23)	
Senior high school	1,611 (26.51)	1,625 (26.55)	
University or college and above	3,178 (52.28)	1,164 (19.02)	
**Marital status**			0.032
Married	5,833 (95.97)	5,919 (96.70)	
Other	245 (4.03)	202 (3.30)	
**Length of time to nearest medical institution**			<0.001
≤ 15min	5,211 (85.74)	5,089 (83.14)	
>15min	867 (14.26)	1,032 (16.86)	
**Health Records**			<0.001
Yes	3,748 (61.67)	2,068 (33.79)	
No	2,330 (38.33)	4,053 (66.21)	
**Chronic disease**			0.002
Yes	273 (4.49)	207 (3.38)	
No	5,805 (95.51)	5,914 (96.62)	
**Cognition of basic public health service**			<0.001
Yes	4,651 (76.52)	4,056 (66.26)	
No	1,427 (23.48)	2,065 (33.74)	

### Utilization of Health Education

In this study, the acceptance rates of health education for registered and floating populations were 87.07% (5,292/6,078) and 79.58% (4,871/6,121), respectively, and the difference was statistically significant (*X*^2^ = 123.034, *p* < 0.001). Meanwhile, the acceptance rate of health education for all types of the floating populations was significantly lower than that of the registered population (*p* < 0.001; (see Table 2).

**Table 2:** Comparison of health education utilization between registered residence population and floating population.

**Types of health education**	**Registered population (6,078)**	**Floating population (6,121)**
Occupational disease prevention and control	2,845 (46.81)	2,021 (33.02)
*P*	<0.001
STD and AIDS prevention and control	3,428 (56.40)	2,510 (41.01)
*P*	<0.001
Reproductive health and contraception	4,354 (71.64)	3,655 (59.71)
*P*	<0.001
Tuberculosis prevention and control	2,823 (46.45)	2,072 (33.85)
*P*	<0.001
Tobacco control	3,749 (61.68)	3,191 (52.13)
*P*	<0.001
Mental health	3,051 (50.20)	2,207 (36.06)
*P*	<0.001
Chronic disease prevention and control	3,069 (50.49)	2,251 (36.78)
*P*	<0.001
Maternal and child health care	4,579 (75.34)	3,911 (63.89)
*P*	<0.001
Self-help education in public emergencies	3,545 (58.33)	2,784 (45.48)
*P*	<0.001

*There are overlaps in the number of participants who received the different types of health education*.

### Current Situation of Vaccination for Children

The data of this study showed that 94.39% (5,737/6,078) of children in registered residence were completely vaccinated, which was significantly higher than that of the floating children (91.68%, *X*^2^ = 34.430, *p* < 0.001). Among the registered population, differences in regional classification (*p* < 0.001), ethnicity (*p* < 0.001), an education level (*p* < 0.001), marital status (*p* < 0.001), length of time to the nearest medical institution (*p* < 0.001), and cognition of basic public health service (*p* = 0.026) aspects between completely and incompletely vaccinated children were statistically significant. For the floating population, the analysis showed that there were significant differences in vaccination rates among floating children of different regional classifications, ethnicity, educational levels, marital status, length of time to the nearest medical institution, health records, cognition of basic public health service, and health education (*p* < 0.05; see Table 3).

**Table 3:** Comparison of complete vaccination among children with different demographic characteristics.

**Variables**	**Completely vaccinated**	
	**Registered population (6,078)**	**Floating population (6,121)**
**Region**		
Eastern region	2,680 (97.28)	2,606 (94.39)
Central region	1,558 (92.19)	1,244 (91.61)
Western region	1,499 (91.79)	1,762 (88.01)
*P*	<0.001	<0.001
**Gender**		
Male	2,964 (94.52)	2,850 (92.02)
Female	2,773 (94.26)	2,762 (91.34)
*P*	0.660	0.329
**Age (years)**		
≤ 30	2,345 (94.40)	2,184 (92.50)
>30	3,392 (94.38)	3,428 (91.17)
*P*	0.967	0.066
**Ethnicity**		
Han ethnic	5,068 (95.33)	5,015 (92.24)
Minorities	669 (87.80)	597 (87.28)
*P*	<0.001	<0.001
**Education level**		
Primary school or below	190 (84.07)	628 (84.07)
Junior high school	992 (93.32)	2,352 (90.99)
Senior high school	1,519 (94.29)	1,530 (94.15)
University or college and above	3,036 (95.53)	1,102 (94.67)
*P*	<0.001	<0.001
**Marital status**		
Married	5,531 (94.82)	5,449 (92.06)
Other	206 (84.08)	163 (80.69)
*P*	<0.001	<0.001
**Length of time to nearest medical institution**		
≤ 15min	4,943 (94.86)	4,690 (92.16)
>15min	794 (91.58)	922 (89.34)
*P*	<0.001	0.003
**Health Records**		
Yes	3,554 (94.82)	1,948 (94.20)
No	2,183 (93.69)	3,664 (90.40)
*P*	0.062	<0.001
**Chronic disease**		
Yes	256 (93.77)	189 (91.30)
No	5,481 (94.42)	5,423 (91.70)
*P*	0.650	0.840
**Cognition of basic public health service**		
Yes	4,407 (94.75)	3,748 (92.41)
No	1,330 (93.20)	1,864 (90.27)
*P*	0.026	0.004
**Health Education**		
Yes	4,991 (94.31)	4,489 (92.16)
No	746 (94.91)	1,123 (89.84)
*P*	0.496	0.008

### Analysis of Influencing Factors of Vaccination in Children

To explore the influencing factors of complete vaccination of children in the registered population and floating population, logistic regression analysis was carried out. Sociodemographic variables and basic public health services (such as, health education and health records) were defined as independent variable X, and vaccination of children was defined as dependent variable Y, as shown in Table 4. The findings indicated that region, education level, and marital status were the main influencing factors of children's vaccination, regardless of registered residence, or floating population. Those who live in the East (OR_1_ = 1.546, OR_2_ = 1.834) have a high level of education (OR_1_ = 2.341, OR_2_ = 470), are married (OR_1_ = 2.682, OR_2_ = 2.094), are more likely to have their children fully vaccinated. Besides, ethnicity (OR = 2.219) and length of time to the nearest medical institution (OR = 1.353) were unique risk factors for complete vaccination of children in registered residence. For the floating population, health records (OR = 1.745) were a unique influencing factor for children's vaccination.

**Table 4:** Logistic regression analysis of the influencing factors of complete vaccination.

**Variables**	**Registered population**		**Floating population**	
	***OR***	***95% CI***	***OR***	***95% CI***
**Region**				
Western region	1		1	
Eastern region	1.546*	1.066–2.243	1.834***	1.453–2.316
Central region	0.542***	0.386–0.758	1.095	0.845–1.418
**Ethnicity**				
Minorities	1		1	
Han ethnic	2.219***	1.561–3.154	1.149	0.871–1.514
**Education level**				
Primary school or below	1		1	
Junior high school	1.818*	1.151–2.872	1.543**	1.199–1.986
Senior high school	1.921**	1.219–3.026	2.347***	1.727–3.189
University or college and above	2.341***	1.506–3.637	2.470***	1.753–3.480
**Marital status**				
Other	1		1	
Married	2.682***	1.831–3.930	2.094***	1.440–3.045
Length of time to nearest medical institution				
>15min	1		1	
≤ 15min	1.353*	1.022–1.791	1.227	0.976–1.542
**Health Records**				
No	1		1	
Yes	1.159	0.899–1.495	1.745***	1.385–2.197
**Cognition of basic public health service**				
No	1		1	
Yes	1.128	0.845–1.505	0.986	0.801–1.214
**Health Education**				
No	1		1	
Yes	0.843	0.586–1.214	1.150	0.918–1.440

*p <0.05, **p <0.01, ***p <0.001.

## Discussion

As an important labor force in urban development, the floating population has made great contributions to promote the rapid development of social economy. However, influenced by the household registration system and other related welfare systems, the floating population cannot enjoy the same public service and social welfare as the registered population (17). Under the condition of low economic income and lack of medical security and basic medical and health service supply, the health of floating children is particularly vulnerable. Vaccination is an effective method to prevent infectious diseases and is considered one of the most cost-effective public health services for children (5, 18, 19). This study takes children vaccination as the breakthrough point and analyzes the differences in the utilization of public health services between the registered residence population and the floating population, provide a theoretical basis for promoting health equity.

### Analysis of Vaccination Status in Children

In China, planned immunization for children began in 1978. At present, the goal of reaching 85% of children's immunization rate has been achieved. Yet, with the rapid increase of the floating population, the immunization planning and management of floating children have become the focus of current work (20). Our research showed that 94.39% of children in registered residence were completely vaccinated and only 5.61% were incompletely vaccinated, but the proportion of incomplete vaccination among the floating children had reached 8.32%. These results were similar to others from diverse population groups. A study in southern Ethiopia found that compared with children born to non-migrant mothers, children born to rural-rural migrant mothers had significantly less chance of receiving full immunization coverage (21). Kagoné et al. conducted a qualitative study in Burkina Faso and also reported that migration was an important reason for incomplete vaccination (22). It can be seen that although China has made some achievements in immunization, the vaccination status of floating children still needs to be further improved. Addressing this issue will be of high significance to the goal of achieving a 100% vaccination rate among children in China.

### Influencing Factors of Immunization Among Children

Univariate analysis showed that regardless of the registered or floating population, regional classification, ethnicity, education level, marital status, length of time to the nearest medical institution, and cognition of basic public health service were related to complete vaccination of children. Parents in the eastern region, Han nationality, college degree or above, married, closer to medical service institutions, and familiar with basic public health services have a higher complete vaccination rate for their children. In addition, the difference in the utilization of basic public health services also has a significant impact on the vaccination of children of the floating population. The complete vaccination rate of children of the floating population who establish health records and understand basic public health services was higher. However, this result seems somewhat less significant in the registered population. According to logistic regression analysis, there were also differences in influencing factors of complete vaccination of children between the two groups.

#### Region

It is widely known that the economy of Eastern China is relatively developed, followed by the central region. Affected by many factors, such as history, society, and natural conditions, the economic development of the western region is at the lowest level in the country. Empirical evidence suggests that large inequity in resources and services can exacerbate disparities in health outcomes and quality of life (23, 24). As this study found, the possibility of children to be vaccinated completely in the East was 1.546 times that of the Western registered population, and among the floating population, this multiple reached 1.834. This may be related to the regional differences in the distribution of health resources. On the one hand, the eastern developed region has a higher level of resources than the other two regions (25). On the other hand, a higher economic level means that the eastern provinces have a higher financial capacity to fund health services. Besides, the larger size and the higher density of populations in the eastern region mean that its operational costs of health services are relatively cheaper (26). Obviously, the western region lacks such resource advantages, which suggests that policymakers should focus on the current situation of health resource allocation in the western region and give corresponding policy and financial support.

#### Ethnicity

The current study found that ethnicity was an influencing factor for complete vaccination of children in the registered population. The probability of complete vaccination of the Han population was 2.219 times higher than that of ethnic minorities. Previous studies have shown that under the influence of Confucian traditional culture and the specific culture of ethnic minorities, the health consciousness of ethnic minorities is relatively limited (27). At the same time, some ethnic minorities trust their traditional treatment methods more, which leads to poor awareness of children's vaccination services or eligibility for vaccines free of charge by ethnic minority parents. This suggests that health education and publicity activities should be implemented for this population, especially the knowledge of children's vaccination services. However, this result has not been found in the floating population.

#### Educational Level

Results from our study also revealed that the educational level of parents is a significant factor that influences the uptake of complete vaccination among children, which is consistent with the previous research results (4, 28). The higher the educational level of parents, the higher the possibility of complete vaccination of their children. This result is applicable to both the registered population and the floating population. In general, highly educated people usually have higher cognitive level and health awareness (29). Parents with higher education levels are more likely to be better educated on immunization and have a good understanding of the value of completely vaccinating their children as compared to those with primary school and below. On the contrary, the less educated people tend to have poor economic status and weak health awareness and are more likely to make health risk behaviors (30), such as refusing or failing to vaccinate their children on time. This problem is more serious among the floating population, which is similar to the results of some studies. Previous studies indicated that increasing education level of the parents, especially for mothers, can improve the full immunization coverage among floating children (4, 31, 32). Indeed, the higher the education level, the stronger the individual's awareness of self-health management. However, this survey found that the education level of most floating populations was junior middle school or below. Due to mobility, they are not familiar with the process of child vaccination. Therefore, this group is the focus of health education intervention for social workers. In addition, the marital status of parents was associated with the complete vaccination of children. This shows that parents with harmonious family relations are more likely to pay attention to their children's health and complete the vaccination on time. Therefore, attention should be paid to those children whose parents are not around when carrying out health education, especially floating children.

#### Length of Time to the Nearest Medical Institution

For the registered population, length of time to the nearest medical institution was another unique risk factor for complete vaccination of children. Analysis shows that the closer the medical and health institutions, the higher the possibility to complete vaccination for children, which is related to the availability of medical services. As an important part of public service facilities, the accessibility of medical institutions reflects the opportunity and convenience of public access to medical services (33). Obviously, residents closer to medical institutions are more likely to have access to health services. However, this factor does not apply to the floating population. Limited by their own economic ability and the nature of their work, the floating population has less opportunity to consider the accessibility of medical services when a choosing residence. Therefore, there is little difference in the overall accessibility of medical services among this group (34).

#### Health Record

Besides, health record was an independent influencing factor for the vaccination of children of the floating population. For the floating population with health records, the complete vaccination of their children is 1.745 times higher than that without records. In China, the Ministry of Health launched the national health record program in 2009. The establishment of the health record is not only an important means to improve residents' health level but also the primary link to realize the equalization of basic public health services. For the floating population, the establishment of health records is one of the most directly beneficial public health services (35). However, due to regional mobility, most of the floating population knows little about relevant health services. Compared with those who have not established health records, the documented floating population has more opportunities to obtain health information. Therefore, they have more opportunities to learn about children's vaccination. This shows that the health publicity for the floating population needs to be further improved.

#### Health Education

The current study also found that health education had a significant impact on the vaccination of floating children in univariate analysis. According to behavior change theory, individuals with sufficient knowledge and positive attitudes could result in good practice (36). Therefore, accepting and understanding health knowledge and applying it to practice is a complete process of behavior change. Health education is the first step to realize behavior change, i.e., imparting health knowledge. For example, health knowledge lectures can enhance people's understanding of infectious diseases and help people to establish a correct concept of health and improve personal health literacy. Improving personal health literacy will help to further improve health outcomes (37). Thus, for the floating population, receiving health education is not only beneficial to their own health but also conducive to the social stability of the inflow area (38). In this study, although some floating population received health education, they did not receive complete vaccination for their children. This suggests that the publicity and education of planned immunization need to be further improved in order to make the floating population realize the importance of planned immunization to children's health.

## Conclusion

Compared with other common public health intervention, vaccination makes good economic sense and meets the need to care for the weakest members of societies. This study found that compared with the registered population, the floating population is at a disadvantage in using basic public health services. There are still 8.32% of floating children were incompletely vaccinated. The region, parents' education level, and marital status were found to be significant risk factors for complete vaccination of children regardless of the registered or floating population. In addition, ethnicity and length of time to the nearest medical institution were unique risk factors for complete vaccination of children in registered residence. In addition, health record was an independent influencing factor for the vaccination of children of the floating population. The findings of this study have certain reference value for further improving the planned immunization management system. Based on the above factors, policy makers should take targeted policies and measures, such as establishing various platforms for basic public health services for the floating population, to ensure that they can make more convenient and equitable use of public health services.

## Limitations

There are several limitations to this study. Among the limitations is the questions' subjectivity, such as the dependent variable and the possibility of recall bias. Secondly, a cross-sectional survey cannot be determined the time-effect and causality accurately compared with the cohort study, so our study only reveals the correlation between factors. In addition, other factors, such as family economic status, vaccine safety, and vaccine hesitance, should be further explored in future studies.

## Data Availability Statement

The datasets presented in this study can be found in online repositories. The names of the repository/repositories and accession number(s) can be found at: http://www.ldrk.org.cn/.

## Ethics Statement

Written informed consent was obtained from the individual(s) for the publication of any potentially identifiable images or data included in this article.

## Author Contributions

JC designed this study, participated in its implementation, and served as the lead writer. YXi and YXu did the data interpretation and co-wrote the article. GJ helped collect the data and research the literature. JX helped with formatting of this manuscript. All authors contributed to the article and approved the submitted version.

## Funding

This work was supported and funded by the National Key Research and Development Program of China (Grant No. 2021YFC2301603).

## Conflict of Interest

The authors declare that the research was conducted in the absence of any commercial or financial relationships that could be construed as a potential conflict of interest.

## Publisher's Note

All claims expressed in this article are solely those of the authors and do not necessarily represent those of their affiliated organizations, or those of the publisher, the editors and the reviewers. Any product that may be evaluated in this article, or claim that may be made by its manufacturer, is not guaranteed or endorsed by the publisher.
